# My friends, I’m #SOTALLYTOBER: A longitudinal examination of college students’ drinking, friends’ approval of drinking, and Facebook alcohol-related posts

**DOI:** 10.1177/2055207619845449

**Published:** 2019-05-06

**Authors:** Mai-Ly N. Steers, Clayton Neighbors, Robert E. Wickham, Whitney E. Petit, Bradley Kerr, Megan A. Moreno

**Affiliations:** 1 Department of Psychology, University of Houston, United States of America; 2 Department of Psychology, Palo Alto University, United States of America; 3 Department of Pediatrics, University of Wisconsin at Madison, United States of America

**Keywords:** Social media, alcohol, peer influences, college students

## Abstract

**Background:**

Literature has consistently shown a positive relationship between young adults’ social media alcohol-related posts and drinking outcomes; however, the reasons for this association and the psychosocial influences behind students’ posting of alcohol-related content are still unclear. Peer influences have been robustly shown to predict students’ drinking such that students’ perceptions of their friends’ drinking is positively associated with their own drinking.

**Objective:**

Although research has demonstrated that online and offline peer influences are robust predictors of drinking among college students, perceptions of friends’ approval and students’ drinking in relation to alcohol-related posting have yet to be explored longitudinally.

**Methods:**

The current multi-site, multi-method study examined students (*N*=316; 58.7% female) from a Midwest (58.8%) and Northwest university over a 4-year period. All Facebook alcohol-related posts were coded each academic calendar year and perceived friends’ approval of drinking and students’ alcohol use were assessed annually. A lagged, random coefficients negative binomial model was specified to examine between- and within-person effects.

**Results:**

After controlling for perceptions of friends’ alcohol-related posts, results revealed that time, drinking more, and perceiving friends as more approving of drinking were significantly and positively associated with posting alcohol-related content at the between-person level. Moreover, a significant interaction of Time X Drinking, with drinking at the between-person level, emerged such that heavier drinkers tended to post less often over time.

**Conclusions:**

Increases in alcohol-related content posts are likely to over-inflate students’ drinking norms and their drinking. Thus, it is plausible that social media networks containing more alcohol-related content may contribute to cyclical increases in drinking for individuals within that network.

## Introduction

In 2016, Monitoring the Future reported that college students had a higher prevalence (32%) of occasions of heavy drinking (five or more drinks in a row in the past 2 weeks) than their non-collegiate peers (29%).^1^ Relatedly, over 40% of students have reported being intoxicated in the past 30 days, compared to 30% of non-collegiate respondents the same age. This is concerning in that more than double the number of college students (∼20%)^2^ meet criteria for alcohol use disorder as compared with the general population (8%).^3^ Moreover, alcohol consumption can lead to other serious consequences; approximately 696,000 students between 18 and 24 years old have reported getting into a physical altercation at least once while under the influence of alcohol, and 97,000 same-aged students indicate they have been sexually assaulted or raped.^4^ In fact, drinking among this age group is such a serious public health concern that the National Institute on Alcohol Abuse and Alcoholism established an Underage and College Drinking Research Program whose primary mission is to understand “the factors that compel youth to begin drinking, continue drinking, and progress to harmful use, abuse, and dependence”.^5^

## Peer influences on drinking

One traditional psychosocial factor on college students’ drinking is social influence from peers. Borsari and Carey^6^ explored three ways in which peers can influence college students’ drinking: (a) actively attempting to get their peers to drink (direct peer influence); (b) modeling drinking behavior (indirect peer influence); and (c) via perceived drinking norms (indirect peer influence); all three of which have been linked to increased alcohol consumption.^7^ Feedback incorporating perceived and actual peer drinking norms have been utilized successfully in personalized normative feedback interventions, one of the most empirically supported individual-level interventions, to reduce drinking among heavy-drinking college students; thus, a large body of literature spanning decades has been dedicated to exploring how perceived drinking norms are linked to consumption among this group.^8–12^

In general, research has found that students perceived even their closest friends as drinking more (a descriptive norm),^13–18^ and being more approving of heavy drinking (an injunctive norm)^19–21^ than them. Furthermore, these normative perceptions of friends’ alcohol use have been found to be strongly associated with students’ own drinking.^14,18,22^ Some researchers have described the transition from adolescence to adulthood as a “window of vulnerability”^23^ in which peers, especially early on in college,^24,25^ exert more of an influence with respect to changes in health behaviors, such as drinking, than parents, as adolescents leave home and, consequently, take control of their own health behaviors. Most people exhibit a curvilinear relationship with respect to alcohol use in which they begin drinking in adolescence, increase their consumption into their early twenties, and then taper off on drinking as they transition to adulthood;^26,27^ however, some individuals begin a life-long pattern of alcohol abuse during this critical period.^28^ Thus, it may be important to examine the trajectories of peer influence on students throughout their college years and beyond to uncover the psychosocial factors that may be contributing to sustained substance abuse.

### The linkage between social media posts and drinking

Emerging research has suggested that another major source of social influence in the digital age is social media. Due to its worldwide presence (1.47 billion users visit the Facebook daily),^29^ a number of studies have focused on how content on Facebook can influence behaviors. For instance, large-scale experimental studies have demonstrated that content users see on Facebook can lead them to create more positive or negative content on the platform due to emotional contagion.^30^

Consequently, over the last decade, alcohol researchers have explored the influence of users’ alcohol-related social media posts on their drinking. Traditional sources of mainstream media (e.g. television, movies) have been dubbed the “super peer” because promotion or exposure to alcohol-related content on these mediums may surpass that of normal peer influences,^31^ and has been linked to a rise in the initiation of drinking and increased consumption worldwide.^32,33^ Given that social networking sites synergistically combine both peer influences and interactive media, they may be a more predominate and formidable “super peer” than more traditional forms of media.

Thus far, due to its pervasiveness and popularity, most of the research in this domain has focused on alcohol-related content posted to Facebook. Studies have found that users’ Facebook alcohol-related content at time 1, prior to college, directly predicted drinking a year into college.^34^ Moreover, users’ alcohol-related content predicted their frequency and quantity of alcohol use,^35^ and greater intentions to share, “like,” and comment on alcohol-related content status updates were related to greater intentions to drink, especially if the status updates already possessed a large number of “likes” and shares.^36^ Overall, a major, robust finding from the literature is that greater frequency of alcohol-related posts are positively associated with increased drinking and alcohol-related consequences among college students across all social media platforms;^37–39^ however, the underlying reasons and the directionality for these associations are still unclear.

One possible explanation for the association between frequency of alcohol-related posts in predicting drinking is that students may be merely posting about what they are engaging in at the moment. For instance, if they are drinking with friends at a party, they might naturally and organically post to social media about it. That is, college students’ own drinking might directly predict their posting behavior. Another plausible reason for this association may be that peers are influencing students’ alcohol-related posting behavior. Hence, college students might view their friends as being more approving of drinking (e.g. a positive, injunctive norm), which, in turn, influences them to post more alcohol-related content to social media and, consequently, drink more. Finally, it is possible that the combination of peer influences and participants’ own drinking might be affecting their alcohol-related posting behavior.

### The current study

To date, most of the research has examined the positive association between alcohol-related content posts and drinking; however, to our knowledge, there are no long-term studies in the literature that have provided strong evidence as to the directionality of drinking and posting behavior. Moreover, few studies have investigated the reasons that drive participants to post alcohol-related content to Facebook. Thus, this prospective cohort, multi-site, multi-method study was conducted to disentangle the complex relationship between alcohol-related posts and drinking by investigating possible psychosocial factors behind posting alcohol-related content. Specifically, we examined how perceptions of friends’ approval of drinking and students’ drinking may have affected college students’ decisions to post frequent alcohol-related content to Facebook longitudinally over time. Given the nature of our repeated-measures design, where students’ responses/alcohol-related content posts were nested within-person, we utilized a lagged, random coefficients negative binomial model to examine both the between- and within-person levels across a 4-year period.

Based on literature indicating that students tend conform with others less over time,^40^ we expected a significant main effect of time such that it would be negatively associated with posting alcohol-related content (H1). Moreover, in line with extant research, we expected significant main effects both at the between- and within-person levels such that, across the sample, students’ drinking would be positively associated with posting alcohol-related content to Facebook more frequently (H2a),^34–36^ and individual students who drank more relative to their mean would post more alcohol-related content (H2b). Relatedly, we expected that higher friends’ approval would be positively associated with frequency of alcohol-related content posts, both at the between- and within-person levels such that, overall, people who perceived their friends to be more approving of drinking would post more alcohol-related content (H3a), and students who perceived increases in friends’ approval of drinking relative to their mean would post more alcohol-related content (H3b).^14,18,22^ Furthermore, we expected friends’ approval to be a moderator of the association between drinking and alcohol-related content posting both at the between- and within-person level. We predicted that in general, heavier-drinking students who perceived their friends to be more approving of drinking would also be more likely to post the most alcohol-related content to Facebook (H4a); additionally, students who drank more frequently and perceived their friends to be more approving of drinking relative to their individual drinking and friends’ approval means would be more likely to post more alcohol-related content (H4b). Based on literature that suggests college students tend to mature as they take on more traditional adult roles and responsibilities,^41,42^ leading to a decrease in drinking over time,^43,44^ we expected a significant interaction between time and drinking, with drinking at the between-person level, such that the tendency for heavier drinkers to post more alcohol-related content would diminish over time relative to lighter drinkers (H5). Finally, we expected a significant three-way interaction between time, drinking, and perceptions of friends’ approval at the between-person level to emerge, such that heavier-drinking lower classmen who perceived their friends to be more approving of drinking would post alcohol-related content more frequently as compared to heavier-drinking upper classmen who also perceived their friends as being highly approving of drinking (H6).

#### Methods

##### Participants and procedure

This study was completed between 2010–2015. The two relevant Institutional Review Boards provided approval. Recruitment targeted graduated high school seniors committed to attending one of two large Midwestern or Northwestern public universities. Eligible participants were 17 to 19 years old and enrolled as full-time, first-year students. Students who arrived on campus during the summer for early-enrollment programs were excluded, as baseline measures were intended to capture pre-college experiences. Approximately 600 students randomly selected from registrars’ lists of first-year students comprised this study’s potential participants. The study included 316 participants, of whom 58.7% were female and 58.8% were from the Midwestern university. Mean age at enrollment was 17.9 years.

Recruitment began with a pre-announcement postcard. Over 4 weeks, potentially eligible students received up to four rounds of emails, phone calls, and Facebook messages. Interested students participated in informed consent conversations over the phone. Researchers informed potential participants that the study would involve both phone interviews and evaluation of Facebook profiles. Thus, participants were asked to “friend” a laboratory Facebook account. It was stressed that although profiles would be viewed to gain a sense of health information present on Facebook, no researcher would add or endorse any content to a participant’s Facebook timeline. Participants were given $35 incentives in the first year and the incentives were increased in $5 increments each year. Payments were distributed each year following completion of the phone interview. Because the main predictor variable of drinking used in this study was first administered after students’ sophomore year of college, the predictor variables represented in this study are from sophomore year to 1-year post-baccalaureate.

### Measures

All measures included in the study were assessed via phone annually at the end of each academic year across the 4 years, except for the outcome variable of alcohol-related posts, which was derived via manual coding of participants’ Facebook pages monthly by research assistants each year during the academic calendar year.

*Perceptions of friends’ approval of drinking.* Perceptions of friends’ approval of drinking was assessed via one item: “What percentage of your friends approve the use of alcohol?” (0–100% scale; intraclass correlation coefficient absolute agreement (ICC_AA_ = .75).

*Perception of friends’ alcohol-related content posts to Facebook.* Perceptions of the percentage of friends who post alcohol-related content to Facebook was assessed using the following question: “What percentage of your Facebook friends display or post alcohol references on Facebook (for example, posting pictures of themselves drinking or status updates describing drinking experiences)?” (0–100% scale; ICC_AA_= .84).

*Drinking.* Participants’ drinking was assessed via the Alcohol Use Disorders Identification Test (AUDIT-C) scale, an instrument with three multiple choice questions validated for evaluation of alcohol use among college students.^45–47^ The three items are: “How often do you have a drink containing alcohol?” (“Never” to “Four or more times a week” on a 0–4 scale); “How many standard drinks containing alcohol do you have on a typical day?” (“One or two” to “10 or more” on a 0–4 scale); and “How often do you have six or more drinks on one occasion?” (“Never” to “Daily or almost daily” on a 0–4 scale). The summed score of these items was calculated for each participant (α =.71–.83 for the scale across the 4 years; ICC_AA_= .92).

*Alcohol-related content posts to Facebook*. A total of seven research assistants evaluated and manually coded participants’ Facebook profiles to identify alcohol-related content each academic year (e.g. summer vacations were excluded), using a well-validated, existing codebook described in previous studies.^48,49^ Four main sections of Facebook profiles were systematically coded on a monthly basis: (a) the About section (e.g. “I may be drunk, Miss, but in the morning I’ll be sober, and you will still be ugly” listed as a favorite quote); (b) the “likes” area, which included pages created by organizations or other interest groups endorsed by the participants, (e.g. “beer pong”); (c) the timeline section, including status updates, wall posts, comments, or “likes” generated by a participant or their friends (e.g. a link posted by a participant about how to make strawberry margarita Jell-O shots); and (d) the photo section, including the profile and cover photos, as well as other images posted by the participants or their friends (e.g. tagged picture of a participant holding a shot glass to their mouth).

Alcohol references were defined according to the Theory of Reasoned Action and were included if they represented an attitude, intention, or behavior toward alcohol.^50^ The codebook was designed to be conservative; if a potential reference did not make explicit mention of alcohol, it was not included. For text-based content, such as was identified in the About, likes, or timeline sections, an explicit mention of alcohol-related terminology or context was needed for a post to be included as an alcohol reference. Therefore, text-based posts using the term “drink” or “party” were not included unless a separate, explicit mention of alcohol was present. Photo-based content was included if a participant was in an image holding, or within arm's length of, an alcoholic beverage. A beverage was determined to be an alcoholic beverage if 1) beer was visible inside; 2) a label indicating the type of beverage was visible; or 3) an alcohol-specific beverage glass, such as a Moscow mule glass, was used. Therefore, for example, a red solo cup whose contents were not visible was excluded.

Each incidence of alcohol-related content was given a count score of one and these scores were summed at the end of each of the 4 years to create the outcome variable of alcohol-related content posts for that particular year. A 20% random subsample of profiles underwent evaluation by all coders as a test of interrater reliability. Fleiss’ κ was used as a measure of overall agreement in the coding of the presence or absence of alcohol-related content on a profile. Over the 4 years assessed during the course of the study, the κ statistic ranged from 0.78–0.82, indicating substantial agreement.

### Plan of analysis

Given the repeated measures design, data were analyzed using multilevel modeling. The outcome variable, alcohol-related content posts, was a positively skewed count variable. Thus, we utilized a generalized linear mixed model approach (a multilevel, negative binomial model) with a random intercept to account for person-level variance; robust estimations of standard errors, and outcomes were specified as negative binomial with a log link.^51^ Analyses were conducted using the MENBREG procedure in STATA 15/SE^52^ where the predictor variables of drinking and friends’ approval were lagged (e.g. posts at time 2 were predicted by drinking and friends’ approval at time 1; posts at time 3 were predicted by drinking and friends’ approval at time 2).^53^ Between-subject (level 2) predictors included participants’ average drinking across years and their average perceived friends’ approval of drinking. Level 2 predictors were grand-mean centered. Linear changes in posting over time were examined as a function of between-subject variables by the interaction terms for the three predictors (Time X Drinking, Time X Friends’ Approval, Drinking X Friends’ Approval, and Time X Drinking X Friends’ Approval). Time was coded from -1.5, -.5, .5, and 1.5 (for years 1 through 4, respectively). In addition to time, within-subjects (level 1) predictors included participants’ drinking, perceived friends’ approval, and the interaction term (Drinking X Friends’ Approval). Level 1 predictors were mean-centered within person. Analyses controlled for perceived percentage of friends who posted alcohol-related content on Facebook.

## Results

A multivariate random intercept model was used to estimate between- and within-person variance components and correlations for the primary study variables. Between- and within-person correlations and intra-class correlations are presented in Table 1. Intra-class correlation coefficients suggest that approximately 40% of the variance in alcohol-related content posting and perceptions of friends’ approval occurred at the between-person level, whereas more than 70% of the variance in drinking behavior was between-persons. Conversely, approximately 60% of the variance in alcohol-related content posting and perceptions of friends’ approval occurred at the within-person level, whereas less than 30% of the variance in drinking behavior was within-person.

**Table 1.** Between, within, and intra-class correlations among primary study variables.

	2.	3.	4.	SD between
1. Time	−.066*	.092*	.241**	–
2. Alcohol-related content posts	.437***	.419***	.325***	5.950
3. Drinking	.048*	.737***	.515***	2.125
4. Friends’ approval	.087	.277**	.463***	11.409
Mean	5.724	4.159	84.737	
SD within	6.760	12.298	1.269	

Note: Within-person correlations below the diagonal, between-persons above, and intra-class correlations provided along the diagonal. *N*=316, *p <.05, **p <.01, ***p <.001. The mean of alcohol-related content posts represents the average of the count scores of alcohol-related content contained on participants’ Facebook profiles across the 4 years.

The results, including the coefficients and incident rate ratios (IRR), are presented in Table 2. The random intercept was significant, indicating that mean number of alcohol-related posts varied among participants. Our hypothesis that there would be a significant overall main effect for time (H1) was supported. The IRR (i.e. eB) of .690 indicates that number of posts reduced by an average of 31% at each time point. In support of H2a, there was a significant effect of drinking on alcohol-related posts at the between-person level but not at the within-person level (H2b), which indicated that heavy drinkers posted more alcohol-related content. Specifically, as indicated by the IRR (eB = 1.155), the number of alcohol-related content posts increased by 16% for each unit increase in participants’ mean drinking (AUDIT-C) score. However, changes in drinking were not significantly associated with changes in their subsequent posts.

**Table 2.** A lagged random coefficients negative binomial model was run with frequency of alcohol-related content posts as the outcome variable.

	b	SE b	Z	p	eB	eB 95% CI
Intercept	0.996	0.11	9.06	0.000	–	2.183-3.360
Friends’ posts	0.008	0.003	2.81	0.058	1.008	1.002-1.014
Time	−0.371	0.061	−6.08	0.000	0.690	0.613-0.778
Drinking between	0.144	0.046	3.16	0.002	1.155	1.056-1.264
FA between	0.017	0.009	1.93	0.024	1.017	1.000-1.035
Time X Drinking Between	−0.041	0.028	−2.50	0.013	0.933	0.884-0.985
Time X FA Between	0.005	0.005	1.14	0.362	1.005	0.996-1.015
Drinking Between X FA Between	−0.002	0.002	−1.14	0.178	0.998	0.993-1.002
Time X Drinking Between X FA Between	−0.002	0.002	−1.14	0.178	0.998	0.993-1.002
Drinking Within	0.006	0.041	0.14	0.515	1.006	0.928-1.091
Friends FA Within	−0.001	0.005	−0.24	0.789	0.999	0.988-1.009
Drinking Within X FA Within	−0.008	0.004	−1.73	0.224	0.993	0.984-1.001
Lnalpha (dispersion)	−0.417	0.116	−3.59	0.000	0.659	0.525-0.827
Var (Random Intercept)	1.488	0.195	7.62	0.000	4.43	3.022-6.494

Note. *N*=316. Bold lines represent significant coefficients. eB are exponentiated coefficients, which are interpretable as rate ratios.

FA: friends’ approval.

Our hypothesis that perceiving friends as approving of drinking would be associated with posting more alcohol-related content posts was also supported at the between-person (H3a) but not at the within-person level (H3b). At the between-person level (eB = 1.017), participants posted 1.7% more alcohol-related content posts for each unit increase in percentage of friends who approve of drinking. Thus, participants who believed on average that 90% of their friends approved of drinking posted 17% more alcohol-related content relative to participants who believed on average that 80% of their friends approved of drinking. Changes in the perceived percentage of friends who approved of drinking was not prospectively associated with changes in number of alcohol-related content posts from year to year. Friends’ approval of drinking did not moderate associations between drinking and posting of alcohol-related content at either the between- or within-person levels. Thus, no support was found for H4a or H4b.

**Figure 1.**
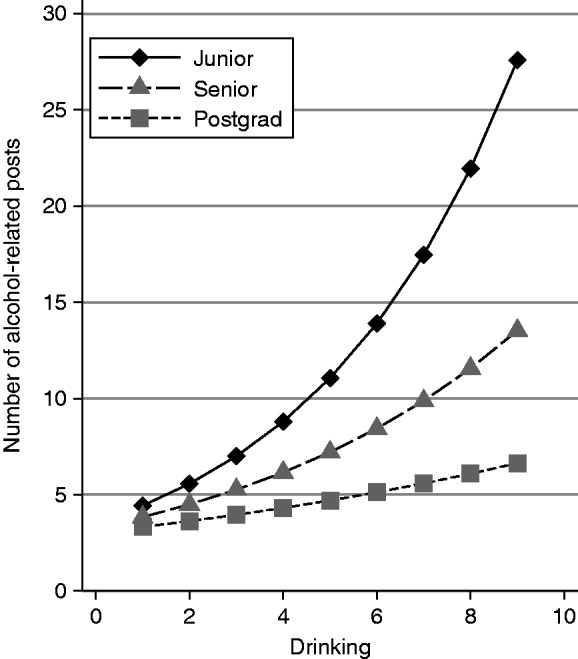
After entering the predictors, controlling for perceived percentage of friends who post alcohol-related posts, the respective two-way interactions, and three-way interaction, a significant interaction between time and drinking at the between-person level emerged in predicting frequency of alcohol-related content posts to Facebook.

There was a significant interaction between time and drinking at the between-person level (H5). Reductions in alcohol-related posts over time were larger among heavier drinkers. As illustrated in Figure 1, heavier drinkers posted substantially more than lighter drinkers early on in college (e.g. during their junior year) but by 1-year post graduation, their posting behavior was similar to that of lighter drinkers. Furthermore, although we did not make specific predictions as to whether frequency of alcohol-related content posts would change as a function of friends’ approval over time (e.g. the Friends’ Approval X Time interaction) at the between-person level, we included the interaction term in the model as a control variable and results revealed this interaction was not significant. Finally, the expected three-way interaction between time, drinking, and perceived friends’ approval of drinking at the between-person level was not supported (H6).

## Discussion

Risky drinking patterns are common throughout college and are related to a variety of negative consequences.^54^ Moreover, one of the most consistent predictors of college students’ drinking is peer influence.^6^ Although there is a robust positive association between posting of alcohol-related content and drinking within the literature,^37–39^ to date, limited efforts have been made to explore the psychosocial factors that might be motivating participants to post such content and investigate how these associations change over time. Thus, the purpose of the present study was to bridge this gap in research and explore psychosocial factors such as drinking and friends’ approval of drinking in relation to posting alcohol-related content on Facebook. The multi-level, lagged structure of our longitudinal, repeated-measures design allowed for in-depth examination of both the between-and within-person effects as well as temporal effects of the predictors on alcohol-related posts.

Overall, we found the number of alcohol-related content posts went down over time but fluctuations in drinking and friends’ approval were not associated with subsequent changes in posting. That is, posts did not increase or decrease following fluctuations in drinking or friends’ approval. By contrast, heavier drinkers posted more alcohol-related content relative to lighter-drinking students and students who perceived their friends as more approving posted more alcohol-related content versus students who perceived their friends as less approving of drinking. Furthermore, reductions in the number of alcohol-related posts over time varied by students drinking such that heavier drinkers who initially posted much more alcohol-related content in their junior year posted less alcohol-related content over time compared to lighter drinkers, who remained relatively stable in their posting of alcohol-related content across the years.

Although previous research has established an association between drinking and posting alcohol- related content, researchers have yet to disentangle whether drinking is driving students to post alcohol-related content or if posting alcohol-related content is influencing drinking. Thus, a major contribution of our study is that it suggests heavier drinkers and students with friends who approve of drinking post more alcohol-related content but that changes in posting behavior do not follow from changes in their drinking or perceptions of peer approval. In addition, posting behavior is less frequent over time, but primarily for heavy drinkers. That is, heavier drinkers tend to post a lot of alcohol-related content earlier on in college but this effect gradually decreases over time to the point that they post about the same as non-heavy drinkers by 1-year post-graduation. However, there could be several possible explanations for these results.

First, researchers^23^ have proposed a “window of vulnerability” model, which suggests that when a person experiences a critical or vulnerable period (e.g. when grown children leave home and live on their own) they are more open to the influence of new social models (e.g. other college students). These “socializing agents” become more impactful for health beliefs and take the place of parental influence as students adjust to a new environment^23^ and begin looking to their peers for cues in determining socially desirable behavior, such as how much to drink at a party.^55–57^

However, in observing their peers and cultivating norms surrounding drinking, students often experience pluralistic ignorance, in which students privately do not condone heavy drinking but participate in heavy drinking anyway because they believe drinking heavily in college is normal.^58^ Thus, students’ misperceptions with regard to how much other students drink often directly impacts students’ own drinking behavior because people naturally behave in ways that are consistent with what they perceive to be normative.^21,59,60^ Similarly, students might also experience pluralistic ignorance regarding posting behaviors. That is, younger college students who are heavier drinkers may be more eager to portray themselves as a drinker on social media, at least early on in college, because they believe portraying themselves in such a way is socially desirable^39,61^ and they want to fit in. As students get older, however, they may be less likely to conform to with others^40^ as their friend groups become more stable. Furthermore, because their established friend groups are likely attending the same social functions, students might be less inclined to post alcohol-related content in an effort to prove their social worth online, even though the heavier drinkers are still engaging in more consumption.

Relatedly, as students mature and transition to more adult jobs and roles,^41,42^ heavier-drinking students might be more censoring of what information they post to social media, and specifically to Facebook. Friends on Facebook are generally derived from a wider social network and may include family, work/school colleagues, and other authority figures. Hence, they might be concerned that employers, other authority figures, or family might form negative impressions of them as a result of seeing such content. Given students have become aware that what they post to Facebook may be heavily scrutinized by others, they may be less inclined to post alcohol-related content to Facebook, particularly as they grow older.

Another possibility for our finding that heavier drinkers are posting less alcohol-related content over time might have little to do with maturation effects but rather is reflective of changes in the social media landscape towards the end of the study. Although Facebook is still one of the most widely used social networking sites among college students, recent evidence suggests that students may be posting alcohol-related content to other platforms, such as Snapchat or Instagram.^62,63^ Reasons for the migration may include the ability to be less self-censoring on these other social media platforms as compared to Facebook, because family members are often not on those platforms, specific features of the of the platforms (e.g. photo filters on Instagram, disappearing content on Snapchat and now on Instagram), being more anonymous (e.g. unlike Facebook profiles, Instagram, Snapchat, and Twitter profiles do not require the user’s offline identity to be necessarily tied to their online personae), and the friend/followers students choose to add to those specific networks (e.g. Snapchat networks allow for direct messaging to smaller, select groups of people and the only way to add a person as a friend is by knowing their username or phone number). Thus, future research should explore alcohol-related content posted to multiple social media platforms to provide a more comprehensive picture of students’ posting behaviors.

### Limitations and future directions

In spite of the major strengths of this study, such as the strong multi-site, multi-method, longitudinal design, the present research does possess a few limitations. First, we solely evaluated Facebook profiles of university students. Thus, our findings may not be generalizable to other populations (e.g. older generations). Also, data collection for the variables examined in this study began in 2011. Facebook was the predominate social networking site used by college students. However, as previously mentioned, recent research suggests students may be migrating to other sites with separate, distinct features. Therefore, just examining Facebook as the sole medium for alcohol-related content may not be sufficient in capturing all alcohol-related content activity posted by college students. Future longitudinal studies could consider incorporating multiple social media platforms as this would also help to determine conclusively whether these effects are the result of a developmental change or a function of our study not encompassing students’ alcohol-related posting behaviors to other social media platforms. Second, we started data collection of the main predictor drinking variables during students’ sophomore rather than freshman year. Moreover, the outcome variable of alcohol-related posts was lagged. Thus, we did not capture how students’ drinking behavior might have been predictive of their posting behavior in their first years of college. It is probable that heavy drinking students may have posted even more content in their freshman and sophomore years relative to their junior and senior years. Third, students could have changed their privacy settings at any point during the study; thus, it unknown whether students altered these settings during the study and to what extent these changes may have resulted in alcohol-related content posts being hidden from the coders. Lastly, we took a conservative approach to coding alcohol-related content. For instance, images that typically demarcate drinking (e.g. solo cups) were not coded unless there was supporting evidence (e.g. such as foam in the cup to indicate beer). However, despite our conservative coding scheme, our findings remained robust.

## Conclusions

This research offers specific implications for researchers and health practitioners by suggesting that interventions related to social media need to be applied to younger, heavier-drinking students, who are potentially more vulnerable to peer influences than older students. Another main implication of the current study and an important future direction to explore is that it suggests the norms distributed via social media may be cyclical, creating a self-perpetuating social media echo chamber that might lead to an over-inflation in drinking and posting norms among that group. Although we controlled for the percentage of friends who post alcohol-related content, we did not assess participants’ perceptions of how frequently their friends posted alcohol-related content to Facebook. Recent research has demonstrated that the frequency of exposure to others' alcohol-related posts was associated with higher consumption and stronger descriptive norms related to online friends’ drinking.^64^

Because it is possible that social media indirectly influences students’ drinking^6^ and consequently their posting behavior, students’ normative perceptions of drinking may become over-inflated due to the frequency of alcohol-related content they view on social media. For example, a given student’s posts of alcohol-related content on social media could be seen by friends or followers within that individual’s social network. Consequently, these postings might be influencing others within the network to post alcohol-related content, which in turn, over-inflates the perceptions of drinking norms, drinking behaviors, and posting of alcohol-related content for all individuals within that network, including those of the original poster.

Thus, exploring how frequently participants see friends’ alcohol-related content and how it relates to participants’ own posting and drinking behaviors may be an important area to explore in the future, because this content may be directly tied to over-inflation of drinking norms. Future longitudinal research should be conducted to examine the frequency in which students see other students’ alcohol-related posts given that frequency may contribute more to students’ normative perceptions surrounding drinking and posting of alcohol-related content than perceptions of the percentage of friends who post alcohol-related content. Another interesting future direction would be to use social network analysis to analyze specific networks of friends to allow for a more comprehensive picture of how drinking relates to posting and vice versa within a particular network. Social network analysis would allow researchers to map and measure the strengths of relationships between people as well as provide both a visual and a mathematical analysis of those relationships.
